# More Significant Impacts From New Particle Formation on Haze Formation During COVID‐19 Lockdown

**DOI:** 10.1029/2020GL091591

**Published:** 2021-04-28

**Authors:** Lizi Tang, Dongjie Shang, Xin Fang, Zhijun Wu, Yanting Qiu, Shiyi Chen, Xin Li, Limin Zeng, Song Guo, Min Hu

**Affiliations:** ^1^ State Key Joint Laboratory of Environmental Simulation and Pollution Control International Joint Laboratory for Regional Pollution Control Ministry of Education (IJRC) College of Environmental Sciences and Engineering Peking University Beijing China; ^2^ Collaborative Innovation Center of Atmospheric Environment and Equipment Technology Nanjing University of Information Science & Technology Nanjing China

## Abstract

During the COVID‐19 lockdown in 2020, large‐scale industrial and transportation emissions were reduced, but high PM_2.5_ concentration still occurred. This study investigated the variation of particle number size distribution during the lockdown, and analyzed the characteristics of new particle formation (NPF) events and its potential impact on haze formation. Through measurement conducted in urban Beijing during the first 3 months of 2020, and comparison with year‐over‐year data, the decrease of primary Aitken‐mode particles was observed. However, frequencies, formation rates and growth rates of NPF events remained stable between 2020 and 2019 in the same period. As a result, >25 nm particles produced by NPF events, would play a more important role in serving as the haze formation “seeds” compared to those produced by primary emissions. This finding emphasizes the significance on the understanding of NPF mechanisms when making pollution mitigation policy in the future.

## Key Points

- Significant reduction of primary Aitken‐mode particles was observed during COVID‐19
- Frequency, formation rates and growth rates of NPF events during the lockdown of 2020 were comparable with those in the same period of 2019
- The “seeds” produced by NPF events would play a more important role in haze formation under the reduction of primary emissions

## 1 Introduction

At the end of 2019, the coronavirus disease (COVID‐19) broke out globally and posed a great threat to public health safety (Morawska et al., 2020). There have been more than 40 million confirmed cases, including more than 1 million deaths until October 31, 2020 (https://covid19.who.int/). Moreover, the epidemic has greatly restricted the development of global economy (Lai et al., 2020). Due to the coronavirus pandemic, many regions implemented a number of lockdown measures to control outdoor activities, leading to reduction of unnecessary emissions. Previous study showed that the cumulative CO_2_ emissions in China during the lockdown declined by 24% (industry), 31% (transport), and 5% (power) compared to the same period of 2019 (Zheng et al., 2020). The primary emissions from industries and transportations are important sources of air pollution, thus improvement of air quality in many countries was reported during the epidemic. NO_2_ decline was the most obvious phenomenon in many regions such as South American, Europe, China, etc. (Huang et al., 2020; Krecl et al., 2020; Sicard et al., 2020). Other pollutants apart from NO_2_ also showed decreasing trends in different levels. About 10%–43% decreases in PM_2.5_, PM_10_, CO, NO_2_ in India were observed during the lockdown compared to 2017–2019 (Sharma et al., 2020). Concentrations of PM_2.5_, NO_x_ and SO_2_ decreased by 15%–61% compared to the periods before COVID‐19 and decreased by 8%–45% compared to 2019 in Yangtze River Delta Region, China (Li et al., 2020). From another viewpoint, COVID‐19 provided an opportunity of experiment on the further emission mitigation. The industry and lifestyle emissions were reduced due to the elasticity of demand, including majority of the transportation (Diamond & Wood, 2020), a small portion of nonpower generation industries, and few of electricity and thermal power generation (Liu et al., 2020), which would be taken as a scenario of future conditions on emission control and air quality improvement.

However, for some cities in northern China like Beijing, there were still significantly heavy pollution processes, and the average concentration of PM_2.5_ experienced an unexpected increase (Le et al., 2020). Many scholars have conducted corresponding researches, and most of them aimed at the chemical and meteorological factors on the haze formation. For instance, Wang, et al. (2020) found that the unfavorable meteorological conditions were the main cause of air pollution, which overwhelmed the benefits of emission reductions. Le et al. (2020) proposed that severe haze events in northern China were formed by aerosol heterogeneous chemistry promoted by high humidity, stable air conditions and uninterrupted emissions from necessary industries. Sun et al. (2020) found higher ratio of secondary organic aerosols (OA) over primary OA, and higher sulfur and nitrogen oxidation capacity in Beijing, indicating stronger secondary chemical transformation during the lockdown.

However, current research lacks the analysis about particle number size distribution (PNSD), which could provide abundant source and secondary formation information. Previous studies on PNSD evolution revealed that in North China, the haze formation usually follow a two‐step pattern: “seeds” (particles with very small diameter, e.g., <100 nm) formation by new particle formation (NPF), and the growth of “seeds” by sufficient secondary aerosol formation (Guo et al., 2014; Shang et al., 2020), especially during fall and winter with relatively higher NPF frequencies (Deng et al., 2020; Wu et al., 2007). Attention should be paid that primary particle emissions can also release those “seeds” into the atmosphere, and their contributions may decrease due to the lockdown. On the other hand, the influence of COVID‐19 on NPF was complicated. As for the sources, the emission reduction may decrease the levels of potential NPF precursors including SO_2_ (Kulmala et al., 2013), NH_3_ (Xiao et al., 2015), Dimethylamine (DMA) (Yao et al., 2018), and VOCs (Fang et al., 2020; Guo et al., 2020), which might restrain the occurrence and intensity of NPF events. Considering the sink, the NPF events may be promoted by the lockdown through the reduction of pre‐existing particles which can act as condensation and coagulation sink of NPF precursors and newly formed clusters (Zhang et al., 2012). Hence, it remains uncertain whether the frequency and intensity of NPF events will increase or decrease during the lockdown. This study focuses on the evolution of PNSD and NPF under the COVID‐19 lockdown, and the results may provide understandings of haze formation not only during the late winter and early spring of 2020, but also in the future condition with cut off of unnecessary industries.

## 2 Materials and Methods

### 2.1 Sampling Station and Measurement Instruments

The measurements were conducted at the Peking University Urban Atmosphere Environment MonitoRing Station (PKUERS) observation site located on the rooftop of a 20‐m‐high building, at the campus of Peking University (39°59′21″N, 116°18′25″E) in the northwest of Beijing. The station represents a typical urban and polluted area with large amounts of fresh, anthropogenic emissions. More detailed descriptions on this measurement site can be found elsewhere (Guo et al., 2014; Wu et al., 2007).

The measurements were conducted from January 1 to March 10, 2020, which covered the periods before and after the breakout of COVID‐19. To exclude the effects of the seasonal variation of NPF events (Deng et al., 2020), the influences of COVID‐19 on PNSD as well as NPF events were investigated by year‐over‐year comparison. Thus, data from the same lunar dates of previous years (2013–2019), especially 2019, was also included (Table S1).

PNSD in the size range of 3–698 nm was obtained by integrating two sets of scanning mobility particle spectrometers (SMPS). The first SMPS measures particles with sizes between 3 and 45 nm, consisting of a TSI Model 3085 DMA and a TSI Model 3776 CPC. The second SMPS measures particles between 45 and 698 nm, consisting of a TSI Model 3081 DMA and a TSI Model 3776 CPC. Detailed procedure can be found in previous study (Zamora et al., 2019). Meteorological parameters, pollutants and photolysis rates, for example, J(O^1^D) were measured at PKUERS site. For a description of the instruments used in this study, see supporting information S4.

### 2.2 Data Analysis of PNSD and NPF

In this study, we characterized a typical NPF event by criteria that PN_3–10_ (particle number concentration in the size range of 3–10 nm) showed an obvious increase (>4,000 cm^−3^) lasting for more than 1 h (Fang et al., 2020). The days without particle formation were defined as non‐NPF days. Other days that failed fulfilling the criteria to be classiﬁed as either NPF event or non‐NPF days were regarded as undefined days. The calculations of formation rate (*J*_3_), growth rate (GR_3‐25_) of 3–25 nm particles and the condensational sink (CS) for sulfuric acid (H_2_SO_4_) were shown in supporting information S1. H_2_SO_4_ is commonly thought to be the most important precursor for nucleation in urban and regional atmosphere (Kuang et al., 2008; Sipilä et al., 2010; Wu et al., 2007). Considering that photochemical oxidation of SO_2_ is the main production process of H_2_SO_4_ (Boy et al., 2005), the concentration of H_2_SO_4_ was estimated with a method using concentration of SO_2_, J(O^1^D) and surface area concentrations of aerosol particles (see supporting information S2). This method was proposed by Zheng et al. (2011), which successfully reproduced the measured H_2_SO_4_ concentrations in summer of Beijing.

## 3 Result and Discussion

### 3.1 Indicating Primary Emission Variation by 25–100 nm Particles

To analyze the influences by COVID‐19, we should first distinguish the COVID‐19 lockdown in 2020 from the lunar New Year influencing period. The lunar New Year is the most important holiday in China, during which around 10 million Beijing drifters and college students left the city, and primary emissions were greatly altered. In this study, a new method is proposed, using variation of 25–100 nm particles number concentrations (PN_25‐100_) to evaluate the primary emission changes in urban environment. The advantage of this method is, compared with tracer gases, 25–100 nm particles are less affected by regional transportation due to their shorter lifetime (Seinfeld & Pandis, 2020), which could mainly reflect local primary emissions.

In general, 25–100 nm particles are correlated with Aitken‐mode particles (diameter within this range), which mainly derived from two processes: one is primary emissions of fossil fuels combustion, and traffic emission is the main source in urban atmosphere; the other one is the condensational growth of <25 nm particles formed by NPF process (Raes et al., 2000; Shen et al., 2011). During winter, the growth of newly formed particles is slower than that in summer due to the weak photochemical reaction under weak radiation (Deng et al., 2020; Wu et al., 2007). Therefore, PN_25–100_ can be used to indicate the intensity of primary “seeds” emissions (traffic emission). The similar diurnal variations between PN_25–100_ and NO_2_ with obvious peaks during morning and evening rush hours confirmed this viewpoint (Figure S1).

**Figure 1:**
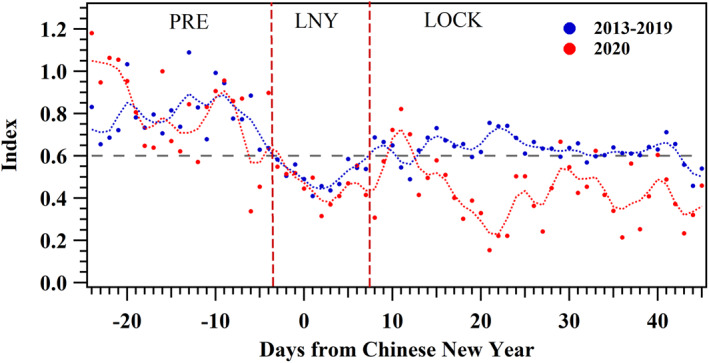
Time series of normalized index of PN_25‐100_ during the same lunar calendar dates in 2013–2019 and 2020. On the *x*‐axis, “0” represents 0:00 on January 1 on lunar calendar (lunar New Year’s Day). The dots and the dotted lines represent the measured and binomial smoothing results in 2013–2019 (blue) and 2020 (red).

Here, we deployed a normalized index of PN_25–100_, calculated through scaling the daily average of PN_25–100_ in each year from 2013 to 2020 with the 95th percentile, to truly reflect the variation of primary emission in the actual urban atmosphere around lunar New Year. The detailed normalized approach is introduced in supporting information S3, and the specific Gregorian calendar dates of used data are presented in Table S1. The higher value means the higher primary emission level. As shown in Figure 1, the normalized index of PN_25–100_ in 2013–2019 was at a high level (around 0.8) before lunar New Year. With the coming of holiday, the index began decreasing and maintained a low degree for several days, then the index rebounded due to the resumption of industry and transportation. The index recovered and maintained a level of 0.6, and it could be regarded as the emission level of normal industrial and transportation activity. Hence, we adopted the level after lunar New Year (index = 0.6) as the judging standard, and took the period from three days before lunar New Year’s Day (−3) to 7 days after lunar New Year’s Day (+7) as LNY (lunar New Year influenced period). It is still worth mentioning that the value after lunar New Year is lower than that before lunar New Year (0.6 vs. 0.8). The reason may be the higher industrial and traffic intensity due to factory rush hours before lunar New Year. As for the index in 2020, it was similar with that in 2013–2019 before and during LNY, but it still stayed a low level after LNY, attributing to the lockdown measures such as the traffic restrictions. Hence, the period after LNY in 2020 was defined as 2020‐LOCK (COVID‐19 lockdown influenced period), and the period before LNY was defined as 2020‐PRE (normal period). The same periods on lunar calendar as 2020‐LOCK and 2020‐PRE in 2019 were defined as 2019‐LOCK and 2019‐PRE (the specific Gregorian calendar dates were presented in Table S2). This distinguishing method could reflect the variation of primary emission in the actual urban atmosphere more truthfully compared with the official holiday division.

### 3.2 Atmospheric Pollutants and Meteorology

Based on distinguishing PRE, LNY, and LOCK periods, changes in meteorological parameters, pollutant emissions and particle number parameters during the three stages in 2020 are present in Figure S2. It can be seen that the heavy pollution episode was not avoided even in 2020‐LOCK on February 9. And the frequency distribution of PM_2.5_ in 2020‐LOCK was similar to that in 2019‐LOCK (Figure S3d). The average concentration of PM_2.5_ in 2020‐LOCK was 67.4 μg m^−3^, which was comparable with that in 2019‐LOCK (73.1 μg m^−3^). This result is consistent with previous research (Huang et al., 2020; Le et al., 2020).

Less NO_2_ and SO_2_ were emitted under the strict lockdown measures. Figure S4 provides the comparison of primary pollutants among 2019‐PRE, 2019‐LOCK, 2020‐PRE, and 2020‐LOCK. The average concentration of NO_2_ in 2020‐LOCK sharply decreased by 59% compared with 2019‐LOCK, and 49% compared with 2020‐PRE, related to the reduction in human activities especially through the traffic restrictions. SO_2,_ a very important precursor of NPF (Wang et al., 2011; Yue et al., 2010) and a marker pollutant for coal burning emission, showed similar patterns to NO_2_. The decreased ratios were 32% and 49% compared to 2019‐LOCK and 2020‐PRE. Considering the essential central heating during winter, the SO_2_ reduction was lower than NO_2_. No significant changes in CO were found in these four stages. BC showed similar variations as PM_2.5_ with concentration in 2020‐LOCK decreased by 24% compared to 2019‐LOCK.

Meteorology is an important factor that can affect air quality. As shown in Figure S3, WS in 2020‐LOCK was relatively higher than that in 2019‐LOCK (2.6 ± 2.0 vs. 1.2 ± 1.6 m s^−1^, with the statistical significance level *p* < 0.001). The temperature in 2020‐LOCK was lower than that in 2019‐LOCK (3.9 ± 4.5 vs. 8.1 ± 6.0°C, *p* < 0.001). Higher RH was observed in 2020‐LOCK compared with 2019‐LOCK (40.7 ± 20.1% vs. 29.0 ± 15.9%, *p* < 0.001). In a word, higher WS and lower temperature were found in 2020‐LOCK compared to 2019‐LOCK in Beijing, which may promote the occurrence of the NPF events (Gong et al., 2010; Yue et al., 2010; Zhu et al., 2013). The relatively higher RH might promote the secondary aerosol production through heterogeneous chemistry (Liu et al., 2017; Sun et al., 2010; Zheng et al., 2015).

### 3.3 The Reduction of the Primary Aerosols Due to the Lockdown

**Figure 2:**
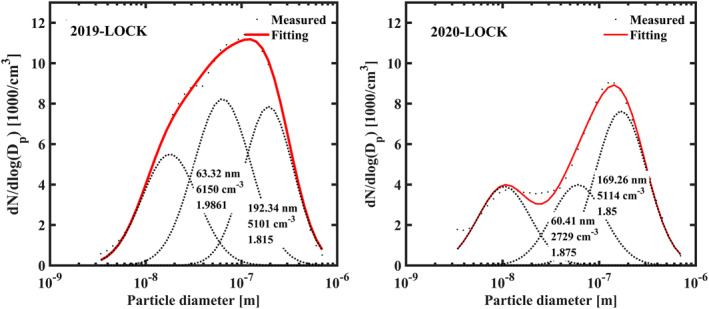
Comparison in mean mode distributions of particle numbers on non‐NPF days at PKUERS in 2019‐LOCK and 2020‐LOCK. The dots represent the measured result and the dotted lines represent the three fitting modes (nucleation mode, Aitken mode and accumulation mode). The red solid lines represent the sum of three fitting modes. The numbers present the geometric mean diameters, total number concentrations, and geometric standard deviations of Aitken mode and accumulation mode.

Fewer primary “seeds” were emitted due to emission reduction. As shown in Figure 2, the average PNSDs during 2019‐LOCK and 2020‐LOCK were fitted as the sum of three mode lognormal distributions (Hussein et al., 2005). By the mean diameter Dp, the fitted modes were interpreted as nucleation mode (Dp < 25 nm), Aitken mode (25 < Dp < 100 nm) and accumulation mode (Dp > 100 nm), respectively. In order to exclude the influence of the NPF process, only non‐NPF days were selected for averaging, of which there were 20 days in 2019‐LOCK and 15 days in 2020‐LOCK. Compared to 2019‐LOCK, number concentration of Aitken‐mode particles (PN_Ait_) sharply decreased by 56% in 2020‐LOCK. This may be attributed to the decreased primary emissions especially traffic emission. Hence, the particle “seeds” for haze formation provided by primary emissions were cut down.

By contrast, the number concentration of Accumulation‐mode particles (PN_acc_) kept even, although the mean diameter decreased from 192 to 169 nm. This result indicates that strict restrictions on primary emissions have not significantly weakened the secondary aerosol production in the atmosphere. Considering that the PN_acc_ are mainly contributed by continuous growth of “seed” particles for 4–7 days (Guo et al., 2014), there could be two reasons for the stability of PN_acc_. The first reason was that the “seeds” from other process, such as NPF were not reduced as much as primary “seeds.” The second reason was that, the intensity of secondary formation process, including photochemical reaction followed by condensational growth or heterogeneous reaction, kept stable or even increased during 2020‐LOCK compared to 2019‐LOCK (Huang et al., 2020; Le et al., 2020), which promoted the growth of “seeds” and added the PN_acc_ in the atmosphere. For example, the heterogeneous reaction in the liquid water content of particles (Shang et al., 2020) may be enhanced by the increasing RH during 2020‐LOCK (Figure S3c).

### 3.4 Increasing “Seeds” for Haze Formation From NPF

**Figure 3:**
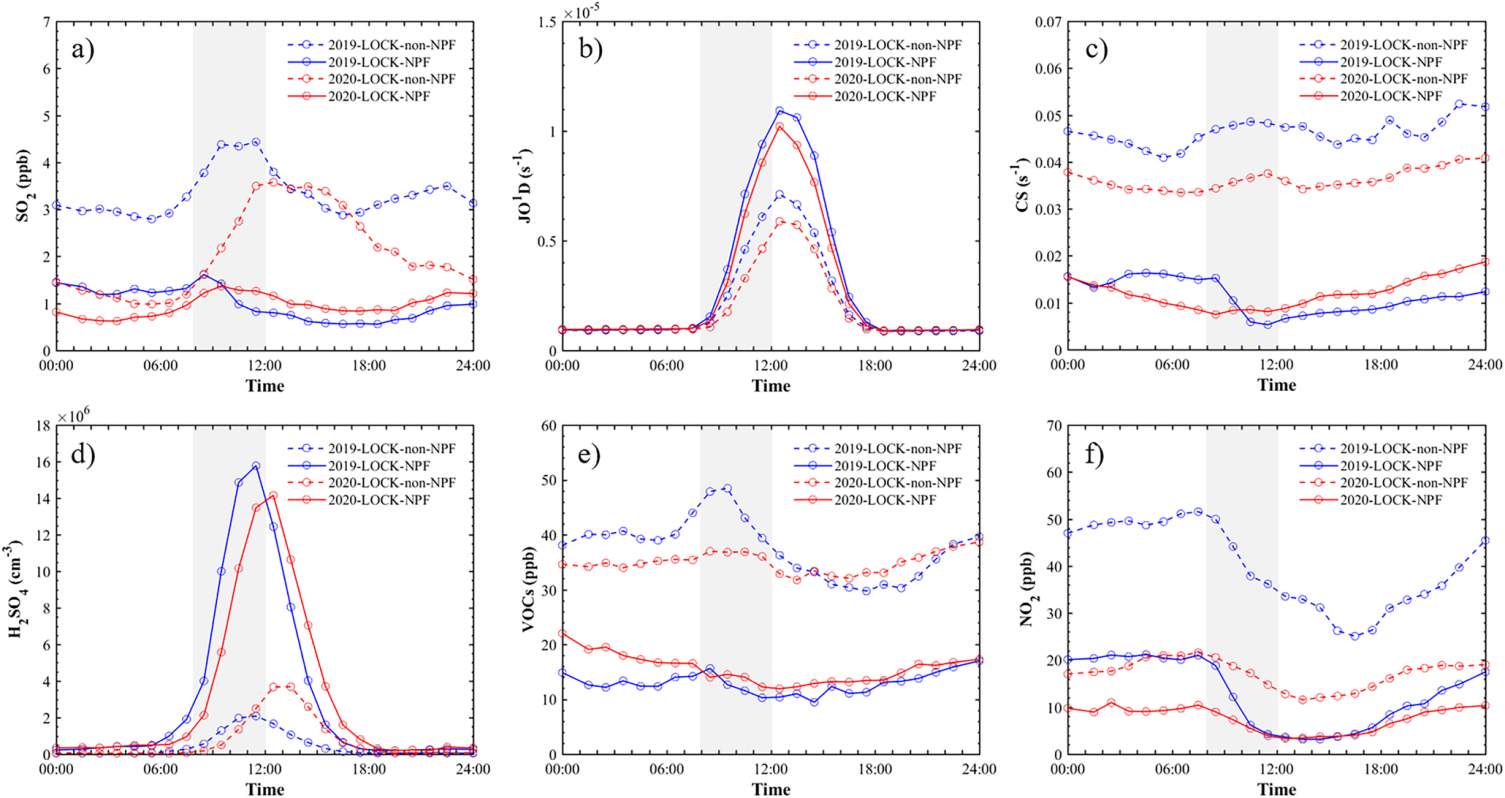
Diurnal variations of mean levels of SO_2_, JO^1^D, CS, H_2_SO_4_, total VOCs and NO_2_ on NPF and non‐NPF days during 2019‐LOCK and 2020‐LOCK. The occurrence time (8:00–12:00) of nucleation is marked by the gray shadow in each panel.

Totally 13 NPF events were observed during 2020‐LOCK, with a similar frequency (36%) as that during 2019‐LOCK (32%). To analyze the influence of the lockdown on the occurrence of NPF events, the diurnal variations of NPF related parameters on non‐NPF days and NPF days during 2019‐LOCK and 2020‐LOCK are compared in Figure 3. The lockdown in 2020 reduced the level of SO_2_ in the morning of non‐NPF days compared with 2019, but the SO_2_ levels were similar during 8:00–12:00 (the occurrence time of NPF events) on NPF days, ranging between 0.6 and 1.6 ppb (Figure 3a). This result indicates that the concentration of SO_2_ on NPF days was mainly controlled by the clean background air masses, and this background condition might not be reduced by the lockdown. Besides, the SO_2_ concentration on NPF days was lower than that on non‐NPF days, implying that this background SO_2_ level is sufficient for nucleation, and do not limit the occurrence of NPF events. In terms of CS, the lockdown had impacts on non‐NPF days. Average CS showed clear decrease from 2019‐LOCK (0.48 s^−1^) to 2020‐LOCK (0.36 s^−1^) in the morning of non‐NPF days, while no clear reduction was found (from 0.01 s^−1^ to 0.009 s^−1^) on NPF days (Figure 3c).

With similar levels of SO_2_, CS and radiation (JO^1^D) (Figure 3b), the calculated concentrations of H_2_SO_4_ are similar on NPF days in 2019‐LOCK and 2020‐LOCK, with a max value of 1.6 × 10^7^ cm^−3^ and 1.4 × 10^7^ cm^−3^, respectively (Figure 3d). This result indicates that source and sink parameters of NPF events during NPF days exhibit more like a background characteristic, which is mainly provided by the meteorological condition and not able to be changed by the emission reductions during the lockdown. Thus, the frequency of NPF events is currently controlled by the occurrence of feasible meteorological conditions (e.g., higher wind speed and elevation of boundary layer), the reduction of primary aerosols and SO_2_ during 2020‐LOCK have little effect on NPF frequency.

**Figure 4:**
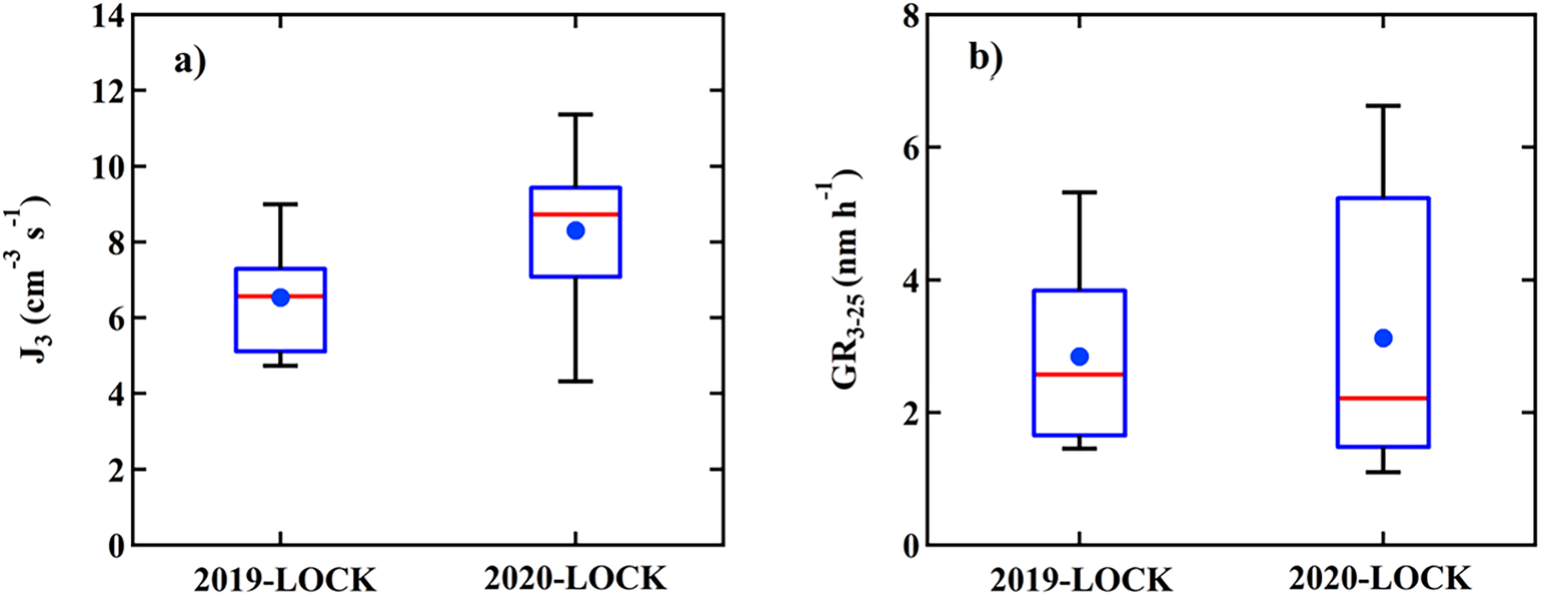
The box‐plots of *J*_3_ and GR_3‐25_ on NPF events during 2019‐LOCK and 2020‐LOCK. The whiskers above and below the boxes are the 90th and 10th percentiles; the upper and lower boundaries of the boxes indicate the 75th and 25th percentiles; the lines and the markers inside the boxes are the median and mean values, respectively.

In terms of the formation rates, the median of *J*_3_ during 2020‐LOCK was higher. The *J*_3_ was 6.5 ± 1.4 cm^−3^ s^−1^ and 9.5 ± 4.0 cm^−3^ s^−1^ during 2019‐LOCK and 2020‐LOCK, respectively (Figure 4a). Since the diurnal variations of H_2_SO_4_ on NPF days was similar (Figure 3d), the higher formation rates in 2020‐LOCK may result from the slightly lower CS in the morning of NPF days (Figure 3c). DMA, NH_3_, organic acids and other nucleation precursors may also influence the nucleation rates, which were not measured in this study.

The newly formed particles with diameter less than 10 nm can hardly impact PM_2.5_ without continuous growth. As shown in Figure 4b, GR_3‐25_ ranged within 1.1–9.7 nm h^−1^ and 1.5–6.0 nm h^−1^ during 2020‐LOCK and 2019‐LOCK, respectively. Those levels are comparable with the previous research in Beijing (Deng et al., 2020). The median of GR_3–25_ showed no remarkable difference between 2020‐LOCK and 2019‐LOCK. However, the mean, 75th percentile and 90th percentile of GR_3‐25_ during 2020‐LOCK are higher than 2019‐LOCK, indicating the faster growth events during 2020‐LOCK. According to previous studies, apart from the condensing of H_2_SO_4_ and HNO_3_ (Wang et al., 2020), highly oxygenated organic molecules (HOMs) formed by oxidation of VOCs (Tröstl et al., 2016), etc. can also participate, or even dominate in the particle growth. The diurnal variations of the mean levels of total VOCs concentrations on NPF days are comparable between 2019‐LOCK and 2020‐LOCK on NPF days, even though their mean values on non‐NPF days are sharply lower in 2020 (Figure 3e). One potential explanation for the higher growth rate events in 2020‐LOCK is that the morning concentration of NO_2_ during 2020‐LOCK is around 50% lower than that during 2019‐LOCK (Figure 3f). NO_2_ is thought to have restraining effect on the formation of HOM monomers and dimers, which can strongly contribute to initial particle growth (Yan et al., 2020). With lower NO_x_, the potential concentration of HOMs can be higher in 2020‐LOCK, enhancing the particle growth.

Since no decrease are shown by both formation and growth rates during 2020‐LOCK, it can be concluded that the number concentration of >25 nm particle “seeds” produced by NPF events were at least not reduced, if not increased, during the lockdown. In addition, the occurrence of NPF events normally spread on large scale of over 100 km in the North China Plain (Shen et al., 2018), which can produce considerably high potential of >25 nm particle, regionally. In contrast, according to the discussion in 3.3, the PN_Ait_ emitted by local fossil fuel combustion, which can serve as “seeds” in haze formation, showed clear downtrend due to the lockdown. Thus, the proportion of particle “seeds” from NPF can be higher under the primary emission reduction, that is the NPF would have a strong impact on the haze formation. For instance, it can be observed that before the occurrence of haze on January 25 and February 9 in 2020, the NPF events produced large number of nanoparticles on January 23 and February 4 (Figures S2f and S2g). In general, the particle number concentration, mean diameter and particle mass concentration followed a 4–9 days cycle, with NPF producing large number of nanoparticles at the start, then PM_2.5_ was promoted by sustained growth of particle diameter (Figures S2c and S2f). A detailed case study on NPF induced haze formation cycle during February 4–14 is provided in supporting information S5, which agreed with the cases reported in previous studies in Beijing (Chu et al., 2021; Guo et al., 2014). These results imply that NPF may be one important cause for the haze events not only during COVID‐19, but also in the future under primary emission control policies.

## 4 Conclusions

As a result of the reduction in primary emissions mainly from the transportations during the COVID‐19 lockdown in 2020 (2020‐LOCK), the concentrations of NO_x_, SO_2_ and PN_Ait_ were sharply decreased compared with the same period in 2019 (2019‐LOCK). However, due to the reduction of preexisting particles and the feasible meteorological conditions such as higher WS and lower temperature, the frequencies of NPF events are comparable between 2020‐LOCK and 2019‐LOCK. On the NPF days, the mean diurnal variations of NPF precursors and radiation showed no remarkable difference between 2020‐LOCK and 2019‐LOCK, leading to similar or even higher formation and growth rates in 2020‐LOCK. Hence, proportion of the NPF‐produced “seeds” increased relative to primary emitted “seeds.” Those “seeds” can induce the secondary aerosol formation through condensational growth and heterogeneous reactions (Shang et al., 2020). Thus, NPF would play a more important role in haze formation. This implication requests more studies on the mechanisms and impacts of NPF events, since the COVID‐19 lockdown may act as a future air quality measure scenario with continuous efforts on primary emission control.

## Conflict of Interest

The authors declare no conflicts of interest relevant to this study.

## Supporting information

Supporting Information S1

## Data Availability

The associated data can be downloaded online (https://doi.org/10.5281/zenodo.4408830).
