# Supplementary material for: More Significant Impacts From New Particle Formation on Haze Formation During COVID‐19 Lockdown
Source: Geophys Res Lett. 2021 Apr 28;48(8):e2020GL091591. doi: 10.1029/2020GL091591 (PMC8206662; doi:10.1029/2020GL091591)
Supplement: Supplementary file 1 — Supporting Information S1 [file GRL-48-e2020GL091591-s001.pdf]

AGU PUBLICATIONS

Geophysical Research Letters

Supporting Information for

More significant impacts from new particle formation on haze formation during COVID-19 lockdown

Lizi Tang[1], Dongjie Shang[1**], Xin Fang[1], Zhijun Wu[1,2], Yanting Qiu[1], Shiyi Chen[1], Xin Li[1,2], Limin Zeng[1,2], Song Guo[1,2], Min Hu[1,2*]

[1]State Key Joint Laboratory of Environmental Simulation and Pollution Control, College of Environmental International Joint Laboratory for Regional Pollution Control, Ministry of Education (IJRC), Sciences and Engineering, Peking University, Beijing 100871, China

[2]Collaborative Innovation Center of Atmospheric Environment and Equipment Technology, Nanjing University of Information Science & Technology, Nanjing 210044, China

*Corresponding author: Min Hu (minhu@pku.edu.cn)

**Tang LZ and Shang DJ contributed equally to this manuscript.

Contents of this file

Text S1 to S5
Figures S1 to S5
Tables S1 to S4

Introduction

This file contains supporting information documenting:

Text S1: Calculation of J_3, $GR_{3\text{-}25}$ and CS

Text S2: Estimation of H_2SO_4

Text S3: The Normalizing approach of index of $PN_{25\text{-}100}$

Text S4: Supplementary instruments

Text S5: Case study on NPF induced haze formation cycle during 4-14 February

Figure S1: Diurnal cycle of NO_2 and PN_{25-100} from 9 February to 10 March 2020

Figure S2: Timeline with some important time nodes and timeseries of meteorological parameters, air pollutants and PNSD at PKUERS

Figure S3: Frequency distributions of WS, RH, temperature and $PM_{2.5}$ at PKUERS

Figure S4: Frequency distributions of NO_2, SO_2, CO and BC at PKUERS

Figure S5. Particle nucleation and growth during haze formation on 4-14 February, 2020

Table S1: The specific time periods of 2013-2020 adopted in this study

Table S2: The specific periods in PRE, LNY and LOCK of 2019 and 2020

Table S3: Information of NPF events in 2019-LOCK and 2020-LOCK

Table S4: Instruments used in this study and the parameters they measured.

Text S1.

The formation rate was calculated using the following formula (Cai et al., 2017):

$$J_{d_k} = \frac{dN_{[d_k,d_u)}}{dt} + \sum_{d_g=d_k}^{d_{u-1}} \sum_{d_i=d_{min}}^{+\infty} \beta_{(i,g)} N_{[d_i,d_{i+1})} N_{[d_g,d_{g+1})} - \frac{1}{2} \sum_{d_g=d_{min}}^{d_{u-1}} \sum_{d_i^3=\max(d_{min}^3, d_k^3-d_{min}^3)}^{d_{i+1}^3+d_{g+1}^3 \le d_u^3} \beta_{(i,g)} N_{[d_i,d_{i+1})} N_{[d_g,d_{g+1})} + n_u \cdot GR_u$$

(1)

Where J_{d_k} is the formation rate of particles at size d_k, and d_k is chosen to be 3 nm in this study. d_u is the upper size bound of the target size range, and 25 nm is adopted for the calculation. $N_{[d_k,d_u)}$ is the total number concentration of particles in the diameters of $[d_k, d_u)$. d_i is the lower bound of each measured size bin, and d_{min} is lowest size limit detected by measuring instrument. $\beta_{(i,g)}$ is the coagulation coefficient for the collision between the particle at size of d_i and the particle at size of d_g. n_u is the particle size distribution function which equals to dN/dd_p, and GR_u is the growth rate at size of d_u. The second term on the right side of the equation (RSE) represents the loss of particles in the size range of $[d_k, d_u)$ due to coagulation scavenging with preexisting clusters or particles (coagulation sink). The third term on the RSE represents the increase of particles in the size range of $[d_k, d_u)$ due to coagulation among smaller clusters or particles (coagulation source). The last term on the RSE is the flux of particles growing up to over size d_u.

The growth rate (GR) was obtained by the mode-fitting method (Dal Maso et al., 2005). The particle number size distribution (PNSD) during NPF event days were fitted as the sum of three-mode lognormal distribution. GR was calculated as the variation of the geometric mean diameter D_m of newly formed mode (3-25 nm) in unit internal (Dal Maso et al., 2005):

$$\mathrm{GR} = \frac{\Delta D_m}{\Delta t}$$

(2)

To evaluate the scavenging effects of preexisting particles on condensable vapors, the condensation sink (CS) was calculated as follow (Dal Maso et al., 2005):

$$\mathrm{CS} = 2\pi \mathrm{D} \sum \beta_m (D_{p,i}) D_{p,i} N_i$$

(3)

where D is the diffusion coefficient of the condensing vapor, β_m is the transition regime correction factor, and $D_{p,i}$ and N_i are the diameter and number concentration in the size class i, respectively.

Text S2.

The H_2SO_4 concentrations on NPF days are calculated with a pseudo steady state method, assuming the OH and SO_2 reaction as the only source and condensation on pre-exiting particles as the only sink, with the formula as (Zheng et al., 2011):

$$P_{GSA} = L_{GSA}$$

(4)

where P_{GSA} and L_{GSA} indicate the production and the loss rate of sulfuric acid on the surface of atmospheric aerosols. P_{GSA} is estimated from:

$$P_{GSA} = k_1[\mathrm{OH}][\mathrm{SO_2}]$$

(5)

where k1 is the reaction coefficient between OH radicals and SO_2, with a value of 1×10-12 cm^3s^{-1}. [SO_2] and [OH] are the levels of reactants in molecules cm-3. L_{GSA} is calculated as follow (Freiberg & Schwartz, 1981):

$$L_{GSA} = [H_2SO_4]\frac{\gamma \cdot S \cdot \bar{v}}{4}$$

(6)

where γ is the uptake coefficient of H_2SO_4 on particle surface, taking a value as 0.73 (Jefferson et al., 1997). S indicates the concentration of particle surface area. $\bar{v}$ is the root mean square velocity of H_2SO_4 molecules. The concentration of OH radicals are estimated by the following formula (Ehhalt & Rohrer, 2000):

$$[\mathrm{OH}] = a(JO^1D)^\alpha(JNO_2)^\beta\frac{b[\mathrm{NO_2}]+1}{c[\mathrm{NO_2}]^2+d[\mathrm{NO_2}]+1}$$

(7)

where α =0.83, β =0.19, a = 4.1×10^9, b=140, c = 0.41 and d = 1.7. NO_2 levels are in unit of ppb.

Text S3.

The normalized index for certain atmospheric pollutant (e.g. PN_{25-100}, NO_2, etc.) is calculated as follows:

a. Calculate the daily average of the pollutant concentration around lunar New Year holiday from 2013 to 2020 (Table S1).

In the main text we chose PN_{25-100}, because 25-100 nm particles are mainly derived from primary emission (traffic emission), and have short atmospheric lifetime, as discussed in the manuscript.

b. Get the normalized index of PN_{25-100} through scaling the daily average of PN_{25-100} in each year with the 95th percentile as follow:

$$I_{d,y} = \frac{a_{d,y}}{a_{y,95\text{th}}}$$

(8)

where y is the year, ranged from 2013 to 2020, d is the day from lunar New Year's day, ranged from -24 to +45 (-24, +45). $I_{d,y}$ indicates the index of PN_{25-100} in the d day of y year. $a_{d,y}$ is the daily average of PN_{25-100} in the d day of y year. $a_{y,95\text{th}}$ represents the 95th percentile of $a_{d,y}$ in y year.

The purpose of this step is to remove the weight of absolute concentration of each year. For example, the primary aerosol emission may be weaker in 2019 compared to that in 2013 in Beijing, due to the Air Pollution Prevention and Control Action Plan (2013-2017). The purpose of using the 95th percentile other than max value is to exclude the influence of special days such as strong atmospheric nucleation and growth days.

c. Average the normalized indexes for days with the same lunar dates among 2013-2019 and get the time series of averaged normalized index of PN_{25-100} of 2013-2019 ($I_{d,2013-2019}$) as follow:

$$I_{d,2013-2019} = \frac{\sum_{y=2013}^{2019} I_{d,y}}{7}$$

(9)

The purpose of this step is to reduce the meteorological and weekday-weekend effects, and reflect a common variation of daily average of PN_{25-100} around lunar New Year holiday.

d. Compare the normalized index between 2020 and the average of 2013-2019.

e. Distinguish the PRE (period before LNY), LNY and LOCK (period after the emission activities back to normal level, named as "LOCK" because it is the period when COVID-19 lockdown measures have clear impacts in 2020) periods by time series of averaged normalized index of PN_{25-100} in 2013-2019, as described in main text of manuscript.

The sketch processes of the calculation are shown below:

1) Sketch table of the daily average values.

*d**	$a_{d,2013}$	$a_{d,2014}$	$a_{d,2015}$	$a_{d,2016}$	$a_{d,2017}$	$a_{d,2018}$	$a_{d,2019}$	$a_{d,2020}$
-24	$a_{-24,2013}$	$a_{-24,2014}$	$a_{-24,2015}$	$a_{-24,2016}$	$a_{-24,2017}$	$a_{-24,2018}$	$a_{-24,2019}$	$a_{-24,2020}$
-23	$a_{-23,2013}$	$a_{-23,2014}$	$a_{-23,2015}$	$a_{-23,2016}$	$a_{-23,2017}$	$a_{-23,2018}$	$a_{-23,2019}$	$a_{-23,2020}$
…	…	…	…	…	…	…	…	…
+44	$a_{+44,2013}$	$a_{+44,2014}$	$a_{+44,2015}$	$a_{+44,2016}$	$a_{+44,2016}$	$a_{+44,2018}$	$a_{+44,2019}$	$a_{+44,2020}$
+45	$a_{+45,2013}$	$a_{+45,2014}$	$a_{+45,2015}$	$a_{+45,2016}$	$a_{+45,2017}$	$a_{+45,2018}$	$a_{+45,2019}$	$a_{+45,2020}$

* *d* means day from lunar New Year's day

2) Sketch table for finding the 95th percentile in each year, $a_{y,95th} = Percentile\ (a_{d,y}, 0.95)$*

$a_{y,95th}$	$a_{2013,95th}$	$a_{2014,95th}$	$a_{2015,95th}$	$a_{2016,95th}$	$a_{2017,95th}$	$a_{2018,95th}$	$a_{2019,95th}$	$a_{2020,95th}$
Formula	$Percentile\ (a_{d,2013}, 0.95)$	$Percentile\ (a_{d,2014}, 0.95)$	$Percentile\ (a_{d,2015}, 0.95)$	$Percentile\ (a_{d,2016}, 0.95)$	$Percentile\ (a_{d,2017}, 0.95)$	$Percentile\ (a_{d,2019}, 0.95)$	$Percentile\ (a_{d,2010}, 0.95)$	$Percentile\ (a_{d,2020}, 0.95)$

**d* means the day from lunar New Year's day, y is the year

3) Sketch table for calculating $I_{d,2013-2019}$ and $I_{d,2020}$.

*d**	$I_{d,2013}$	$I_{d,2014}$	$I_{d,2015}$	$I_{d,2016}$	$I_{d,2017}$	$I_{d,2018}$	$I_{d,2019}$	$I_{d,2013-2019}$	$I_{d,2020}$
-24	$\frac{a_{-24,2013}}{a_{2013,95th}}$	$\frac{a_{-24,2014}}{a_{2014,95th}}$	$\frac{a_{-24,2015}}{a_{2015,95th}}$	$\frac{a_{-24,2016}}{a_{2016,95th}}$	$\frac{a_{-24,2017}}{a_{2013,95th}}$	$\frac{a_{-24,2018}}{a_{2018,95th}}$	$\frac{a_{-24,2019}}{a_{2019,95th}}$	$\frac{\sum_{y=2013}^{2019} I_{-24,y}}{7}$	$\frac{a_{-24,2020}}{a_{2020,95th}}$
-23	$\frac{a_{-23,2013}}{a_{2013,95th}}$	$\frac{a_{-23,2014}}{a_{2014,95th}}$	$\frac{a_{-23,2015}}{a_{2015,95th}}$	$\frac{a_{-23,2016}}{a_{2016,95th}}$	$\frac{a_{-23,2017}}{a_{2017,95th}}$	$\frac{a_{-23,2018}}{a_{2018,95th}}$	$\frac{a_{-23,2019}}{a_{2019,95th}}$	$\frac{\sum_{y=2013}^{2019} I_{-23,y}}{7}$	$\frac{a_{-23,2020}}{a_{2020,95th}}$
…									
+44	$\frac{a_{+44,2013}}{a_{2013,95th}}$	$\frac{a_{+44,2014}}{a_{2014,95th}}$	$\frac{a_{+44,2015}}{a_{2015,95th}}$	$\frac{a_{+44,2016}}{a_{2016,95th}}$	$\frac{a_{+44,2017}}{a_{2017,95th}}$	$\frac{a_{+44,2018}}{a_{2018,95th}}$	$\frac{a_{+44,2019}}{a_{2019,95th}}$	$\frac{\sum_{y=2013}^{2019} I_{+44,y}}{7}$	$\frac{a_{+44,2020}}{a_{2020,95th}}$
+45	$\frac{a_{+45,2013}}{a_{2013,95th}}$	$\frac{a_{+45,2014}}{a_{2014,95th}}$	$\frac{a_{+45,2015}}{a_{2015,95th}}$	$\frac{a_{+45,2016}}{a_{2016,95th}}$	$\frac{a_{+45,2017}}{a_{2017,95th}}$	$\frac{a_{+45,2018}}{a_{2018,95th}}$	$\frac{a_{+45,2019}}{a_{2019,95th}}$	$\frac{\sum_{y=2013}^{2019} I_{+45,y}}{7}$	$\frac{a_{+45,2020}}{a_{2020,95th}}$

**d* means the day from lunar New Year's day

Text S4.

$PM_{2.5}$ was measured by a tapered element oscillating microbalance (TEOM, 1400a, Thermo, USA) with a $PM_{2.5}$ cyclone inlet (Zamora et al., 2019). Tracer gaseous pollutants were continuously detected by a series online monitoring system manufactured by Thermo Electron Corporation (O_3 (Model 49i), SO_2 (43i-TLE), CO (48i-TLE) and NO-NO_2-NO_x (model 42i-TLE)). Meteorological parameters including wind speed (WS), wind direction (WD), temperature (T) and relative humidity (RH) were measured by the automatic meteorological station (Met one Instrument Inc). The photolysis frequencies of O_3 (JO^1D) and NO_2 (JNO_2) were monitored by a spectroradiometer, following the procedure described by (Wang et al., 2019). 99 types of volatile organic compounds (VOCs) were measured by the online gas chromatography and mass spectrometry (Fang et al., 2020). Black carbon (BC) was measured by Aethalometer (Magee Scientific, model AE31), and the concentration of BC at 880 nm was used in this study to reduce the influence of Brown carbon (Kirchstetter et al., 2004).

Text S5.

During the clean days, the strong nucleation occurred in the morning of 4 and 5 February, and the total particle number concentration increased to around 7×10^4 cm^{-3} (Fig. S2f), which is mainly contributed by the nucleation-mode particles. Then, mean diameter of the nucleated particles was observed to increase continuously in the following 4 days, from ~8 nm to ~90 nm, when $PM_{2.5}$ increased from 30 μg m^{-3} to 90 μg m^{-3} (Fig. S5). The NPF events on 8 February also injected high number concentration of nucleation-mode particles (~4×10^4 cm^{-3}) into the atmosphere of Beijing (Fig. S2f). From 8 February to 11 February, the mean diameter continued growing to over 120 nm, and $PM_{2.5}$ increased to 280 μg m^{-3} by efficient secondary aerosol formation. The mean diameter and mass concentration fluctuated during the transition of haze periods, which may be influenced by local primary emission, wind speed and planet boundary level.

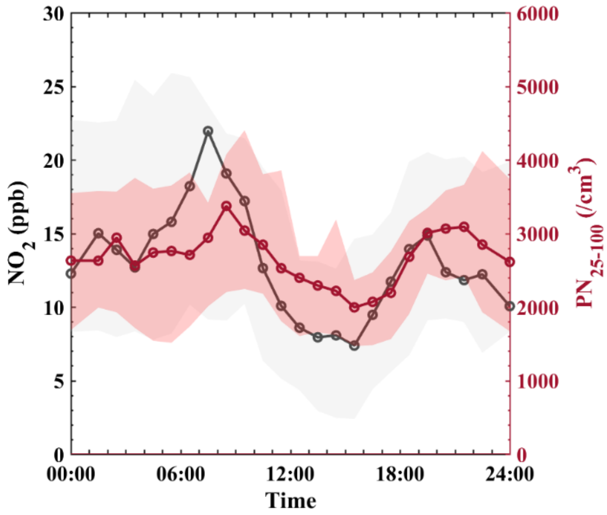

Figure S1. Diurnal cycle of NO_2 and $PN_{25\text{-}100}$ from 9 February to 10 March 2020.

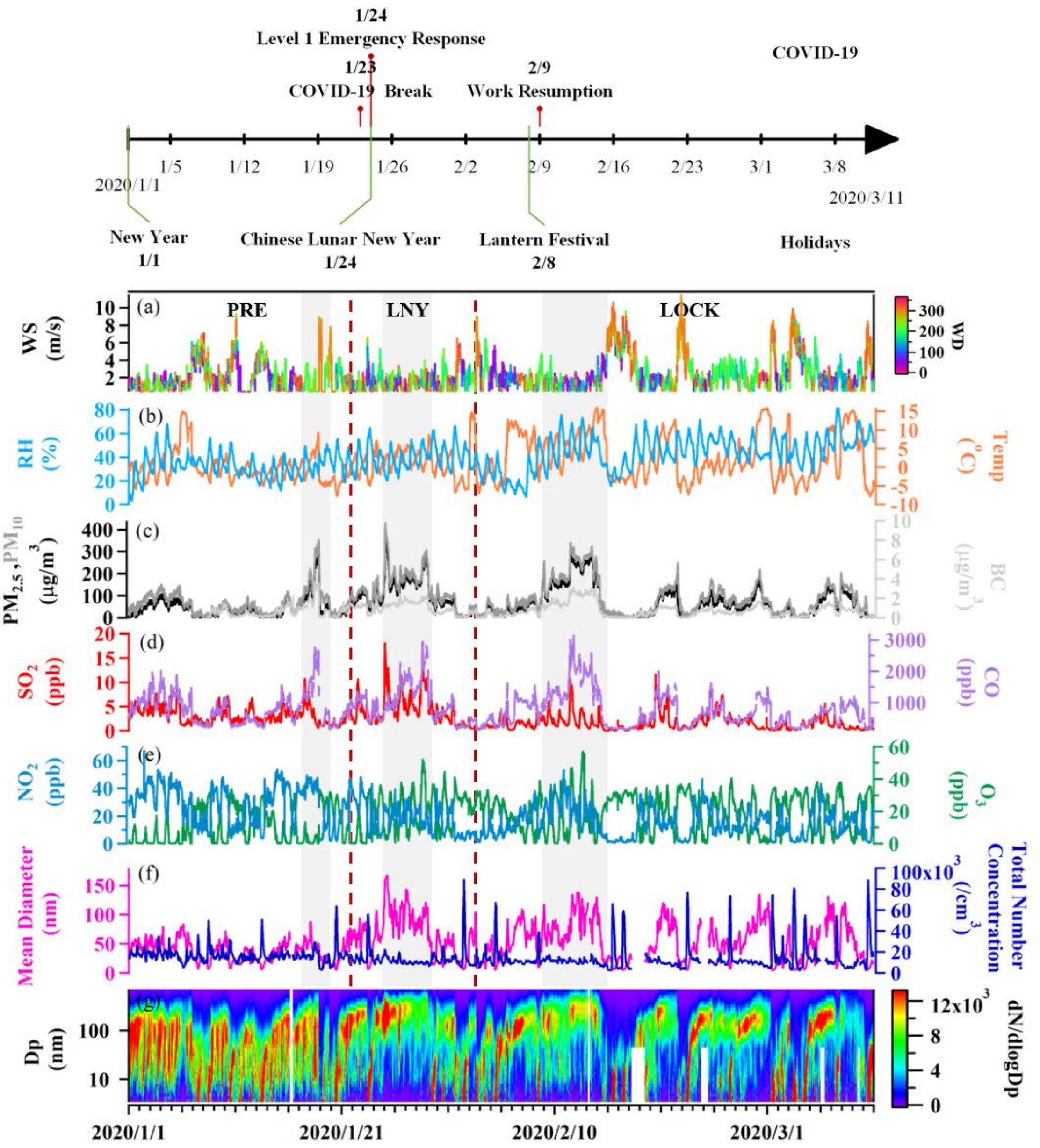

Figure S2. Timeline with some important time nodes from 1 January to 10 March 2020 and timeseries of meteorology parameters, air pollutants and particle number parameters at PKUERS, including (a) WS, WD, (b) RH, temperature, (c) PM_{10}, $PM_{2.5}$, BC, (d) SO_2, CO, (e) O_3, NO_2, (f) mean diameter and total number concentration of particles (ranged from 3 nm to 698 nm), (g) PNSD. The gray background represented 3 heavy pollution episodes which was defined as a process when the mass concentration of $PM_{2.5}$ increased from a level below 50 μg m^{-3} to a peak value exceeding 200 μg m^{-3} and then dropped back to a level below 50 μg m^{-3}.

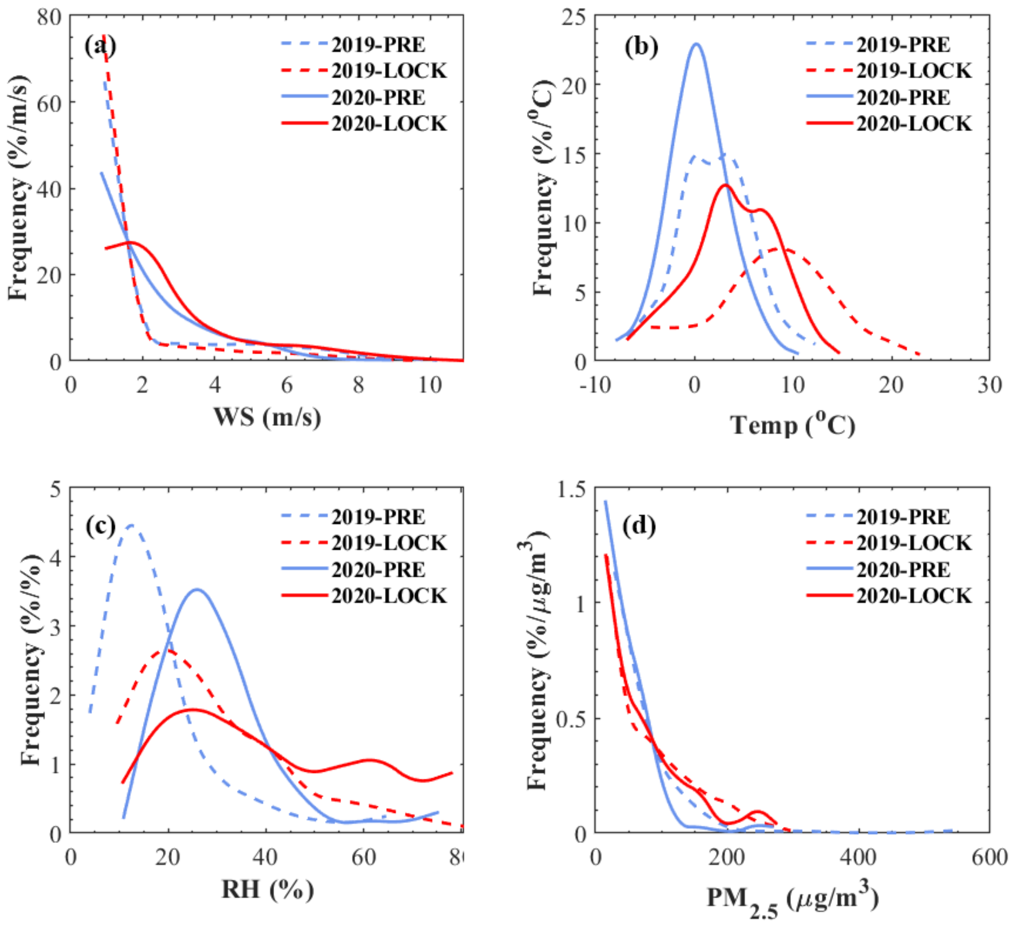

Figure S3. Comparison in frequency distributions of WS, RH, temperature and $PM_{2.5}$ at PKUERS in 2019-PRE, 2019-LOCK, 2020-PRE and 2020-LOCK.

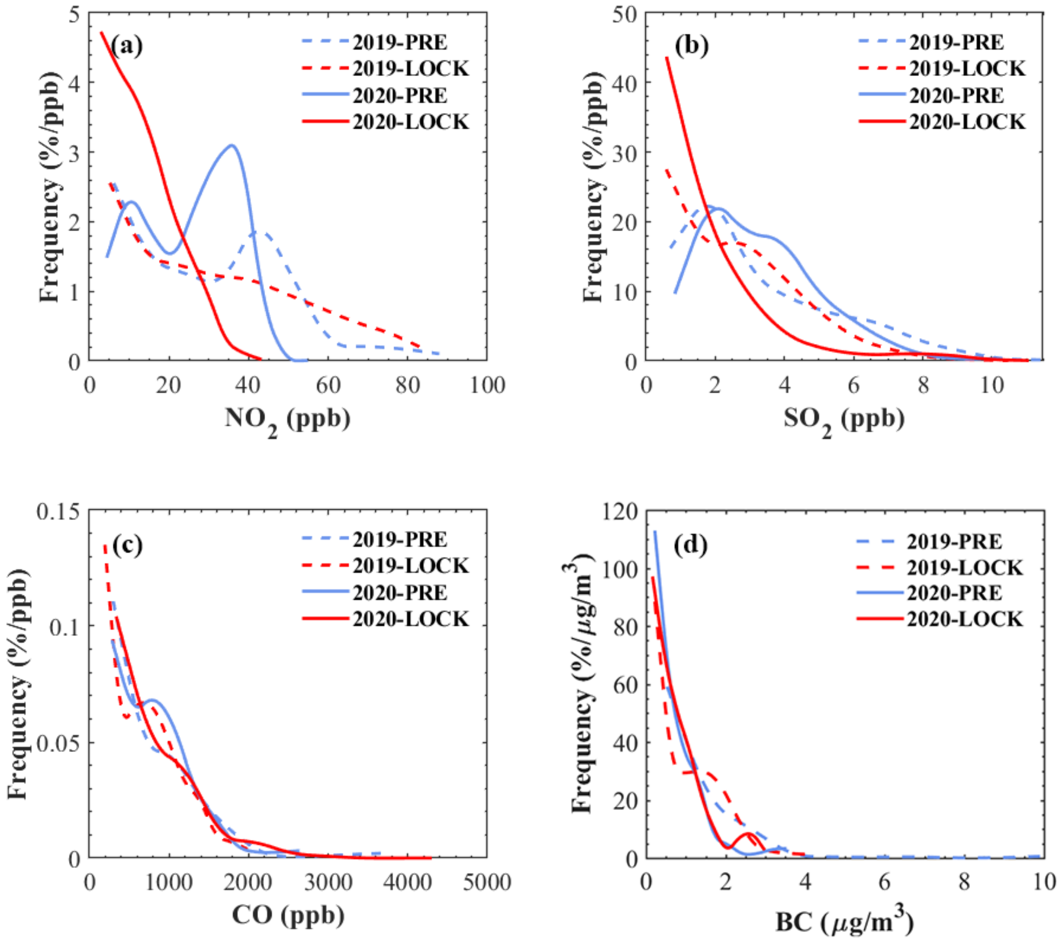

Figure S4. Comparison in frequency distributions of NO_2, SO_2, CO and BC at PKUERS in 2019-PRE, 2019-LOCK, 2020-PRE and 2020-LOCK.

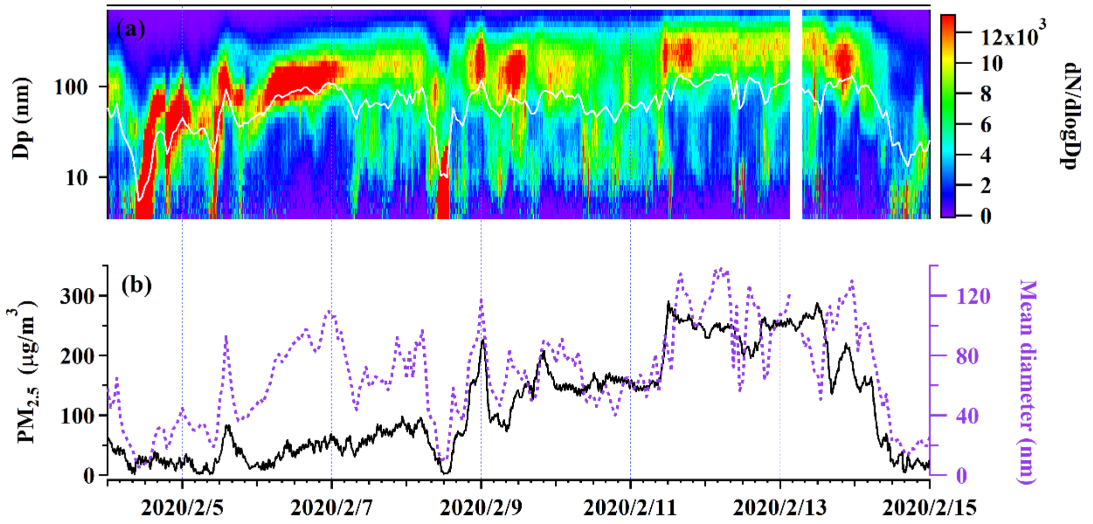

Figure S5. Particle nucleation and growth during haze formation on 4-14 February, 2020. (a) Temporal evolution of PNSD and mean particle (white solid line) on 4-14 February. (b) Temporal evolution of $PM_{2.5}$ and mean diameter on 4-14 February.

YEAR	DATE (-24, +45)
2013	Jan 18th-Mar 28th
2014	Jan 7th-Mar 17th
2015	Jan 26th- Apr 5th
2016	Jan 16th- Mar 25th
2017	Jan 4th-Mar 14th
2018	Jan 23rd-Apr 2nd
2019	Jan 12th-Mar 22nd
2020	Jan 1st-Mar 10th

Table S1. Periods adopted in distinguishing of the periods influenced by COVID-19 and LNY in 2013-2020. The numbers in brackets represent the days from lunar New Year's day.

YEAR	PRE (-24, -4)	LNY (-3, +7)	LOCK (+8, +45)
2019	Jan 12th-Feb 1st	Jan 2nd-Feb 12th	Feb 13th-Mar 22nd
2020	Jan 1st-Jan 21st	Jan 22th-Feb 1st	Feb 2nd-Mar 10th

Table S2. The distinguishing periods in PRE, LNY and LOCK of 2019 and 2020. The numbers in brackets represent the days from lunar New Year's day.

DATE	J_3 ($cm^{-3}s^{-1}$)	GR_{3-25} (nm/h)	CS (s^{-1})	SO_2 (ppb)
20190213	4.9	5.9	0.0052	0.84
20190215	5.2	1.5	0.0038	0.69
20190216	6.1	3.7	0.0051	1.4
20190217	5.1	2.0	0.0051	1.6
20190305	7.2	3.1	0.0079	1.1
20190306	4.7	2.0	0.0075	0.27
20190311	8.2	2.9	0.0055	0.68
20190312	7.3	1.5	0.0056	0.77
20190313	6.8	2.2	0.0065	0.78
20190314	6.4	1.4	0.0067	0.92
20190315	7.2	3.9	0.0080	0.43
20190321	9.3	3.9	0.0065	0.28
20200203	4.1	2.5	0.0081	0.76
20200204	9.4	3.6	0.0050	0.81
20200205	4.7	5.4	0.012	3.2
20200208	10	1.6	0.012	2.8
20200215	7.6	1.2	0.0037	0.24
20200216	7.9	1.3	0.0030	0.44
20200222	8.7	5.4	0.0042	0.58
20200301	9.3	2.2	0.0036	0.25
20200302	6.6	1.8	0.012	1.8
20200303	12	1.1	0.0043	0.33
20200304	9.3	5.1	0.0076	1.2
20200305	9.5	2.0	0.023	2.9
20200310	8.5	7.5	0.0042	0.44

Table S3. J_3, GR_{3-25}, CS and SO_2 during NPF events periods in 2019-LOCK and 2020-LOCK.

Instrument	Manufacturer (country)	Time resolution	Parameters
Meteorological station	Met one (USA)	1 min	WS, WD, Temp, RH
TEOM Model 1400a	Thermo (USA)	1 min	$PM_{2.5}$
Thermo Model 49i	Thermo (USA)	1 min	O_3
Thermo Model 43i-TLE	Thermo (USA)	1 min	SO_2
Thermo Model 48i-TLE	Thermo (USA)	1 min	CO
Thermo Model 42i	Thermo (USA)	1 min	NO_2
Spectroradiometer	PKU (CHN)	1 min	$J(O^1D)$, $J(NO_2)$
Online GC-MS	PKU (CHN)	1 h	Gaseous VOCs
Aethalometer Model AE31	Magee Scientific (CA)	5 min	BC
Nano-SMPS	TSI (USA)	5 min	PNSD of 3–45 nm particles
Long-SMPS	TSI (USA)	5 min	PNSD of 45–698 nm particles

Table S4. Instruments used in this study and the parameters they measured.
